# Limited incremental predictive value of the frailty index and other vulnerability measures from routine care data for mortality risk prediction in older patients with COVID-19 in primary care

**DOI:** 10.1186/s12875-024-02308-5

**Published:** 2024-02-23

**Authors:** Hannah M. la Roi-Teeuw, Kim Luijken, Marieke T. Blom, Jacobijn Gussekloo, Simon P. Mooijaart, Harmke A. Polinder-Bos, Maarten van Smeden, Geert-Jan Geersing, Carline J. van den Dries

**Affiliations:** 1 Department of General Practice and Nursing Science, Julius Center for Health Sciences and Primary Care, University Medical Center Utrecht, Utrecht University, Stratenum 6.131, PO Box 85500, 3508 GA Utrecht, The Netherlands; 2 Department of Epidemiology and Health Economics, Julius Center for Health Sciences and Primary Care, University Medical Center Utrecht, Utrecht University, Utrecht, The Netherlands; 3 Department of General Practice, Amsterdam UMC Location Vrije Universiteit, Amsterdam, The Netherlands; 4 Amsterdam Public Health Research Institute, Amsterdam, The Netherlands; 5 LUMC Center for Medicine for Older People, Department of Public Health and Primary Care, Department of Internal Medicine, Leiden University Medical Center, Leiden, the Netherlands; 6 Section of Gerontology and Geriatrics, Department of Internal Medicine, Leiden University Medical Center, Leiden, The Netherlands; 7 Department of Public Health and Primary Care, Leiden University Medical Center, Leiden, The Netherlands; 8 Section of Geriatric Medicine, Department of Internal Medicine, Erasmus MC, University Medical Center Rotterdam, Rotterdam, The Netherlands; 9 Department of Data Science and Biostatistics, Julius Center for Health Sciences and Primary Care, University Medical Center Utrecht, Utrecht University, Utrecht, The Netherlands

**Keywords:** Frailty, Vulnerability, COVID-19, Older people, Prognosis research, Primary care, Prognostic factor study, Multiple imputation, Regression modelling

## Abstract

**Background:**

During the COVID-19 pandemic, older patients in primary care were triaged based on their frailty or assumed vulnerability for poor outcomes, while evidence on the prognostic value of vulnerability measures in COVID-19 patients in primary care was lacking. Still, knowledge on the role of vulnerability is pivotal in understanding the resilience of older people during acute illness, and hence important for future pandemic preparedness. Therefore, we assessed the predictive value of different routine care-based vulnerability measures in addition to age and sex for 28-day mortality in an older primary care population of patients with COVID-19.

**Methods:**

From primary care medical records using three routinely collected Dutch primary care databases, we included all patients aged 70 years or older with a COVID-19 diagnosis registration in 2020 and 2021. All-cause mortality was predicted using logistic regression based on age and sex only (basic model), and separately adding six vulnerability measures: renal function, cognitive impairment, number of chronic drugs, Charlson Comorbidity Index, Chronic Comorbidity Score, and a Frailty Index. Predictive performance of the basic model and the six vulnerability models was compared in terms of area under the receiver operator characteristic curve (AUC), index of prediction accuracy and the distribution of predicted risks.

**Results:**

Of the 4,065 included patients, 9% died within 28 days after COVID-19 diagnosis. Predicted mortality risk ranged between 7–26% for the basic model including age and sex, changing to 4–41% by addition of comorbidity-based vulnerability measures (Charlson Comorbidity Index, Chronic Comorbidity Score), more reflecting impaired organ functioning. Similarly, the AUC of the basic model slightly increased from 0.69 (95%CI 0.66 – 0.72) to 0.74 (95%CI 0.71 – 0.76) by addition of either of these comorbidity scores. Addition of a Frailty Index, renal function, the number of chronic drugs or cognitive impairment yielded no substantial change in predictions.

**Conclusion:**

In our dataset of older COVID-19 patients in primary care, the 28-day mortality fraction was substantial at 9%. Six different vulnerability measures had little incremental predictive value in addition to age and sex in predicting short-term mortality.

**Supplementary Information:**

The online version contains supplementary material available at 10.1186/s12875-024-02308-5.

## Introduction

The older COVID-19 patients are, the higher their risk of severe illness and mortality. During the pandemic, COVID-19 protection measures (e.g., vaccination) were therefore prioritized to older people. Specific focus was on frail, more vulnerable patients, as this subpopulation was expected to have the worse prognosis [1, 2]. Frailty indicators have indeed been used in several multivariable prediction models for COVID-19 prognosis in hospital settings, besides age, sex, comorbidities, vital signs and laboratory test results [3]. Previous studies confirmed that age and sex are also predictive of COVID-19 mortality amongst primary care patients, with older men having the highest risk [4, 5]. However, whether frailty is predictive for COVID-19 mortality – in addition to age and sex – has never been formally evaluated in a primary care setting.

Nevertheless, rapid identification of older patients with high mortality risk is critical when targeting the inherent limited health care resources and managing patients outside the hospital during a pandemic. It is important to evaluate the prognostic value of frailty in the primary care setting, as the case-mix is considerably different from hospitalized patients. In the Netherlands, the Clinical Frailty Scale (CFS) [6–8] was often used in hospitals for triage based on frailty. The CFS is an ordinal scale with nine categories ranging from very fit (CFS = 1) to frail and terminally ill (CFS = 9), as a subjective assessment that relies on patients’ need for help with basic and instrumental activities of daily living, chronic health conditions, self-appreciation of health and engagement in straining activities. However, this CFS and many other well-known frailty indicators that were used for triage in the COVID-19 pandemic require in-person patient assessments that might be challenging during a pandemic, particularly if the clinician doesn’t know a particular patient well enough. Moreover, even if a frail older patient is well-known to his/her general practitioner, scores like the CFS were not routinely administered and cannot be automatically extracted from routine primary care registries, making the potential selection of older individuals in need for a higher level of care during a pandemic more time-consuming. Of note, important aspects of frailty such as functional status and dependency of others that are commonly included in the assessment of frailty (including the CFS) are difficult to capture in routine care data based on diagnosis codes [9]. Hence, when using measures from routine care as done in this paper, we prefer to speak of ‘vulnerability’ measures.

This study aimed to evaluate the predictive value (in addition to age and sex) of several vulnerability measures using routinely available primary care medical record data, for prediction of 28-day mortality in older patients with COVID-19. We believe acquiring knowledge on the role of such vulnerability measures in COVID-19 prognosis is pivotal, not only to learn from the past COVID-19 pandemic, but also to further explore the resilience of older people during acute illness, thereby being informative for other acute illnesses as well as future pandemic preparedness.

## Methods

The protocol for this study was published online prior to the start of data analysis [10]. Reporting adheres to the transparent reporting of a multivariable prediction model for individual prognosis or diagnosis (TRIPOD) guidelines for prediction model development where appropriate [11].

### Data source

Data were extracted from three routinely collected primary care databases administered by the Julius General Practitioner’s Network (JGPN) University Medical Center Utrecht [12], the Academic Network of General Practice at VU University Medical Center in Amsterdam (ANH VUMC), and the Academic General Practitioner’s Network at Academic Medical Center Amsterdam (AHA AMC) [13]. Data in these databases are derived from electronic medical records of participating local general practices. Raw data include anonymized free-text reports of primary care consultations, using consultation encodings that are used in Dutch primary care medical record systems (diagnostic codes according to the International Classification of Primary Care (ICPC-1) and prescription codes according to Anatomical Therapeutic Chemical (ATC) classification), and other information related to individual consultations and practice registration.

### Participants

In this study, we defined ‘older COVID-19 patients’ as patients aged 70 years or older, in line with other studies [14]. We included all patients aged 70 years or older with a COVID-19 diagnosis registration in 2020 or 2021. During the early pandemic (before 1 June 2020), uniform registration of COVID-19 diagnosis and widespread testing were not established yet in Dutch primary care. For this period, COVID-19 patients were identified by manual free-text screening of consultations that were recorded with the ICPC R74 (unspecified acute upper respiratory tract infection), and by the ICPCs R81 (pneumonia) and R83 (other respiratory infection) – which were ICPC’s GP’s were recommended to use for registration of COVID-19 at the time – amongst patients in JGPN and ANH VUMC. Manual free-text screening was performed by three experienced primary care clinical-researchers. Only patients in whom COVID-19 was mentioned as likely diagnosis in free-text were subsequently included. In case of discrepant judgement, cases were discussed until mutual agreement was reached. More details on the patient selection can be found in a previous publication on this study cohort [4]. From 1 June 2020 up till 31 December 2021, patients were included based on ICPC R83 and R83.03 (COVID-19) in all three databases. If patients had multiple subsequent COVID-19 diagnoses, only the first COVID-19 episode was included in this study. Patients were excluded if their COVID-19 was not diagnosed during active practice registration (i.e. if COVID-19 test results from public health services were linked to the primary care medical record from a period before the patient was registered at the practice, or after the patient left the practice) to ensure data availability and hence mitigate bias from missing data on predictor variables or follow-up.

### Outcome

The outcome was all-cause mortality within 28 days after COVID-19 diagnosis, as registered by either the ICPC A96 (death), practice deregistration due to death, or mention of death in free-text. These include all deaths, regardless whether the patient died at home or elsewhere (e.g., during hospitalization): general practices would always be notified in case of death and deregister the patient, which is strictly monitored by health insurances for patient registration fee disbursement. Free-text of consultations up to 90 days after COVID-19 diagnosis were manually checked to identify delayed death registrations and to confirm the date of death. Patients’ data were analyzed with a follow-up of 28 days after COVID-19 diagnosis, or until death, or until practice deregistration for other reasons, whichever occurred first.

### Predictors

For this study, we have a priori selected six ‘vulnerability measures’ based on their availability in routine primary care data and their *potential* to reflect aspects of frailty according to literature or clinical experience [15, 16]. We selected three composite scores based on cumulative deficits and/or comorbidities: the Frailty Index (FI) from Drubbel and colleagues [17], the Chronic Comorbidity Score (CCS) [18] and the Charlson Comorbidity Index (CCI) [19], all calculated from ICPC- and ATC-codes (see Supplementary Material 1 for more details on these scores). The advantage of these metrics is that they take into account many comorbidities or other aspects of wellbeing and functioning, but a disadvantage is that they require an algorithm for calculation of scores. We therefore also included three simple alternative indicators of vulnerability, that are readily visible in the electronic medical record, namely: the number of chronically prescribed medications (based on ATC-codes, reflecting treatment-requiring conditions), renal function (the estimated glomerular filtration rate (eGFR), reflecting vital organ functioning) or any diagnosis of cognitive impairment (based on ICPC-codes, reflecting biological ageing and care needs). See Supplementary Material 1 for detailed definitions. Predictor data as available on the date of COVID-19 diagnosis were extracted.

### Statistical analysis

Baseline characteristics were described for patients with and without 28-mortality separately, using median and interquartile range (IQR) for continuous non-normally distributed variables. P-values, derived from the Chi-square test or Kruskal–Wallis rank sum test, are not adjusted for multiple testing and hence should be interpreted as indicative of the magnitude of group differences rather than as formal statistical test results.

Missing data on the eGFR were handled by multiple imputation with chained equations (*mice* R package [20]), assuming that eGFR data were missing at random conditional on covariates [21]. We used age (spline function), sex, the interaction between age and sex, the outcome and 29 additional characteristics (amongst which several comorbidities, social economic status, and database) as predictors in the imputation of categorical eGFR (see Supplementary Material 1 for more details).

We defined seven models: a basic model with only age and sex as predictors, and six additional models, each with one of the six vulnerability measures as additional predictor. Each of these models was fitted using unpenalized logistic regression (to obtain model fit parameters), penalized logistic regression (to evaluate apparent performance), and tenfold internal cross-validation of the penalized logistic regression (to evaluate internal performance and internal calibration slope) [22]. Details on the model fitting can be found in the study protocol and are summarized here. [10] All models included interaction terms between sex and age spline terms. Age, FI, CCS, CCI and number of drugs were modelled using restricted cubic spline functions with knots on the 0.05, 0.35, 0.65 and 0.95 percentiles to allow for non-linear effects [23]. Cognitive impairment was modelled as binary variable. We anticipated to model the eGFR as spline function, but due to many qualitative values in the data we decided post hoc to model the eGFR as categorical with seven categories (< 15, 15–30, 30–45, 45–60, 60–75, 75–90, > 90), not exceeding the maximum number of candidate predictor terms according to the sample size calculation. Elastic net penalization (*glmnet* R package, alpha = 0.5) was used to mitigate the risk of overfitting and to enable variable selection, with tenfold cross-validation to determine the value of the shrinkage factor based on deviance optimization [22, 24]. Incremental predictive value was evaluated by comparing model fit parameters, the area under the ROC curve (AUC), index of prediction accuracy (IPA), and the distribution of predicted risks. The IPA, calculated as 1 – (Brier score_model_/Brier score_null model_), represents the percentage improvement in mean sum of squared error of the model compared to the null model (predicting average risk for each patient), thereby incorporating both discrimination and calibration properties [25]. The IPA can best be interpreted relative to other models, with higher IPA indicating a better model, as the absolute value of the IPA is scaled to a hypothetical ‘perfectly accurate’ model (IPA = 100%, zero error) that is typically non-existent. Confidence intervals (95% CI) for the AUC were calculated using the Delong variance estimation method, and for the IPA using a bootstrap sampling distribution from 1000 bootstrap samples. For the models with the eGFR, metrics from ten imputed datasets were pooled using Rubin’s rules (based on a bootstrap standard error for the 95% CI of the IPA).

Two sensitivity analyses were performed post hoc. One analysis restricted the eGFR data to only recent eGFR values recorded within the last five years before COVID-19 diagnosis. Hereto, all eGFR values with older or missing recording date were imputed using the same procedures as for missing eGFR values. Another analysis restricted the study cohort to only individuals with COVID-19 in 2020, before the start of COVID-19 vaccination campaigns, to explore robustness of results over time during the pandemic.

Details on sample size calculations can be found in the Supplementary Material 1. All analyses were performed in R, using the R packages *tableone*, *glmnet*, *rms* and *pROC* [26–30].

### Ethics

The study was waived from formal ethical approval by the Medical Ethics Assessment Committee NedMec (METC NedMec, protocol number 22/857). Informed consent was waived by the Medical Ethics Assessment Committee NedMec (METC NedMec, protocol number 22/857), which declared that the Medical Research Involving Human Subjects Act (WMO) does not apply to this research, as the study only used anonymized data and did not require direct patient or physician involvement. All patients from general practices participating to the JGPN, ANH VUMC and AHA AMC databases can object to the use of their anonymous data in these databases via an opt-out system. This research was conducted in accordance with Dutch law and the European Union General Data Protection Regulation and according to the principles of the Declaration of Helsinki. All methods were carried out in accordance with relevant guidelines and regulations.

## Results

### Study population

**Fig. 1:**
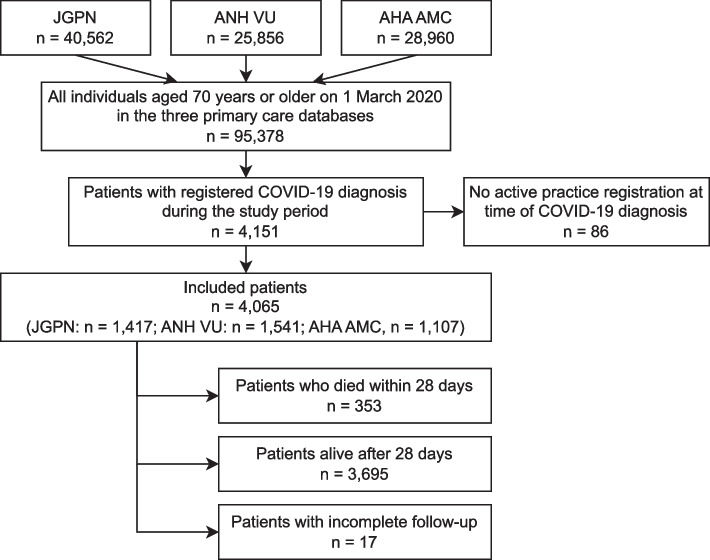
Flowchart of the study population selection

We included 4,065 older COVID-19 patients in this study (Fig. 1). Data were complete for all predictors except for eGFR (*n* = 223 (5.5%) missing). Complete follow-up for 28-days or until death was available for 4,048 patients (99.6%), since 17 patients (0.4%) were practice-deregistered before the end of follow-up, for example when moving to a nursing home. None of these patients moved to a palliative care facility according to the free-text reporting. Therefore, these patients were assumed 28-day survivors for further analyses. In total 8.7% of the study population died within 28 days. Median age was 77 years [IQR: 73 – 83]. Cardiovascular comorbidities were most frequent (71%), followed by diabetes (34%), cancer (28%), chronic kidney disease (26%), lung disease (26%) and cognitive impairment (15%). Half of the study population (52%) had four or more chronically prescribed drugs. FI, CCS and CCI scores were considerably high (Table 1). Characteristics of the study population (Table 1) were similar amongst the three primary care databases (Supplementary Material 1).

**Table 1:** Baseline characteristics of included older COVID-19 patients in primary care

	**Total study population at baseline (*n* = 4,065)**	**Status after 28 days**		
		**Survivors (*n* = 3,712)**	**Deceased patients (***n*** = 353)**	*p*-value^*^
*Demographics*				
Age in years, median [IQR]	77 [73, 83]	76 [73, 82]	82 [76, 88]	< 0.001
Female, n (%)	2185 (53.8)	2046 (55.1)	139 (39.4)	< 0.001
*Comorbidities, n (%)*				
Cardiovascular disease	2898 (71.3)	2609 (70.3)	289 (81.9)	< 0.001
Hypertension	2121 (52.2)	1915 (51.6)	206 (58.4)	0.017
Heart failure	475 (11.7)	402 (10.8)	73 (20.7)	< 0.001
Coronary artery disease	964 (23.7)	868 (23.4)	96 (27.2)	0.123
Cerebrovascular disease	662 (16.3)	574 (15.5)	88 (24.9)	< 0.001
Peripheral artery disease	349 (8.6)	307 (8.3)	42 (11.9)	0.026
Atrial fibrillation	621 (15.3)	544 (14.7)	77 (21.8)	< 0.001
Diabetes	1383 (34.0)	1209 (32.6)	174 (49.3)	< 0.001
Pulmonary disease	1072 (26.4)	952 (25.6)	120 (34.0)	0.001
COPD	493 (12.1)	423 (11.4)	70 (19.8)	< 0.001
Asthma	507 (12.5)	461 (12.4)	46 (13.0)	0.804
Chronic kidney disease	1061 (26.1)	903 (24.3)	158 (44.8)	< 0.001
Liver disease	138 (3.4)	127 (3.4)	11 (3.1)	0.882
Dementia	193 (4.7)	147 (4.0)	46 (13.0)	< 0.001
Immuno-compromised^a^	526 (12.9)	455 (12.3)	71 (20.1)	< 0.001
Cancer	1129 (27.8)	1028 (27.7)	101 (28.6)	0.760
*Vulnerability measures*				
Frailty Index, median [IQR]	0.30 [0.22, 0.38]	0.30 [0.20, 0.38]	0.32 [0.24, 0.40]	< 0.001
CCS, median [IQR]	2 [1, 3]	2 [1, 3]	3 [2, 4]	< 0.001
CCI, median [IQR]	5 [4, 7]	5 [4, 7]	7 [5, 8]	< 0.001
Number of drugs, median [IQR]	4 [1, 7]	4 [1, 7]	5 [2, 9]	< 0.001
eGFR				< 0.001
< 15	18 (0.5)	13 (0.4)	5 (1.5)	
15–30	124 (3.2)	94 (2.7)	30 (8.8)	
30–45	353 (9.2)	296 (8.4)	57 (16.8)	
45–60	746 (19.4)	662 (18.9)	84 (24.8)	
60–75	1221 (31.8)	1137 (32.5)	84 (24.8)	
75–90	1112 (28.9)	1048 (29.9)	64 (18.9)	
> 90	268 (7.0)	253 (7.2)	15 (4.4)	
Cognitive impairment, n (%)	627 (15.4)	528 (14.2)	99 (28.0)	< 0.001

*IQR* interquartile range, *COPD* chronic obstructive pulmonary disease, *eGFR* estimated glomerular filtration rate, *CCS* chronic comorbidity score, *CCI* Charlson comorbidity index

^*^P-values for the comparison between survivors and deceased patients (Chi-square test or Kruskal–Wallis rank sum test) are not adjusted for multiple testing and hence should be interpreted as indicative of the magnitude of group differences rather than as formal statistical test results

^a^According to a diagnosis of immunodeficiency or use of immunosuppressants, including prednisone, biologicals and oncolytics

### Incremental predictive value

**Fig. 2:**
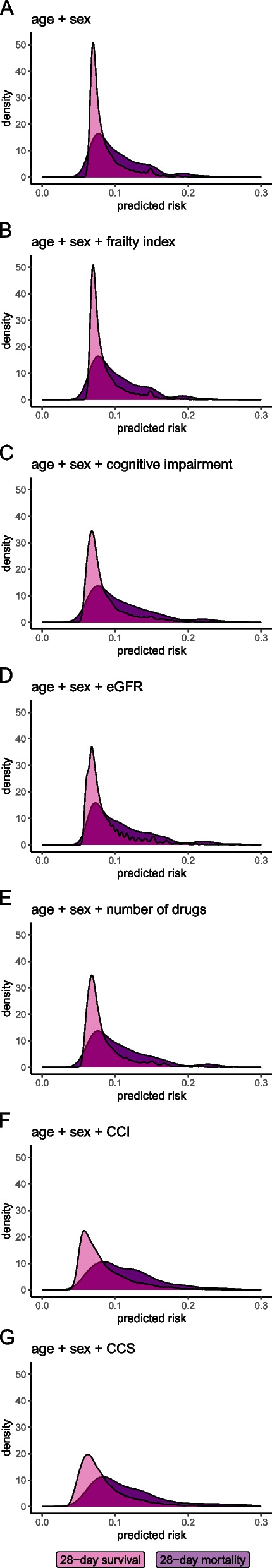
Distributions of predicted risks by penalized models stratified by outcome. Plot A shows the 28-day mortality risks as predicted by the basic model, whereas plots B-G shows the risks predicted by the six vulnerability models, respectively. For each plot, the distribution of predicted risks for patients that survived 28 days is shown in pink, and the distribution of predicted risks for patients that died within 28 days is shown in purple

**Fig. 3:**
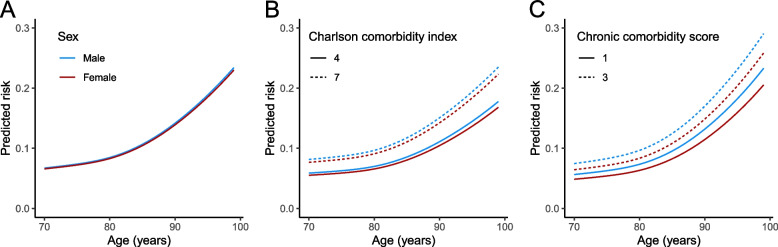
Predicted risks. Plots show the risk that the basic model (plot **A**) model with CCI (plot **B**) or model with CCS (plot **C**) predict, given age (x-axis), sex (colour) and CCI/CCS score (linetype, only for plot B and C). In plot B and C, predicted risks are plotted for the 0.25 and 0.75 percentile values of the CCI and CCS, respectively. CCI = Charlson Comorbidity Index, CCS = Chronic Comorbidity Score

All anticipated models were fitted (see Supplementary Material 1 for coefficients and fit and performance statistics). Predicted 28-day mortality risk ranged between 7 and 26% for the basic model (Fig. 2 and Supplementary Material 1). Notably adding CCS or CCI to the basic model led to a slightly more granularized spread of the distribution of predicted risks, yielding a range between 4 and 41% (Fig. 2 and Supplementary Material 1). Higher predicted risk in males became more apparent after adjustment for comorbidity-based scores (Fig. 3). The basic model had an AUC of 0.69 (95% CI 0.66 – 0.72), which was slightly increased when adding either the number of chronically prescribed medications, cognitive status, or renal function. The highest AUC was reached by addition of the CCI or CCS (both AUC 0.74, 95% CI 0.71 – 0.76). The models with CCI and CCS also had the highest IPA, indicating that not only discrimination but also overall fit improved most for these models (Table 2). A sensitivity analysis on only recent eGFR recordings did not change any of the findings up to two decimals. Baseline characteristics and trends in comparisons between vulnerability models were similar in a subgroup of patients with COVID-19 diagnosis in 2020, thus before the start of widespread COVID-19 vaccination (data not shown).

**Table 2:** Model performance upon internal validation of penalized logistic regression

	Basic (age + sex + age*sex)	**Basic + Frailty index**	**Basic + Cognitive impairment**	**Basic + eGFR**	**Basic + Number of drugs**	**Basic + CCI**	**Basic + CCS**
AUC (95% CI)	0.69 (0.66, 0.72)	0.69 (0.66, 0.72)	0.69 (0.66, 0.72)	0.70 (0.67, 0.73)	0.70 (0.67, 0.73)	0.74 (0.71, 0.76)	0.74 (0.71, 0.76)
IPA, % (95% CI)	3.0 (-5.6, 11.5)	3.4 (-5.1, 11.8)	3.2 (-5.2, 11.7)	3.5 (-7.0, 14.0)	3.8 (-4.5, 12.1)	4.8 (-3.2, 12.8)	5.0 (-3.1, 13.1)

*eGFR* estimated glomerular filtration rate, *CCI* Charlson comorbidity index, *CCS* chronic comorbidity score, *AUC* area under the curve, *CI* confidence interval, *IPA* index of prediction accuracy

## Discussion

### Summary of results

This study evaluated the incremental predictive value of different routine-care based vulnerability measures for short-term mortality in older primary care patients with COVID-19. The 28-day mortality fraction was high (8.7%). A model with only age and sex as predictors yielded an AUC of 0.69. Adding the CCI or CCS as an additional predictor moderately increased model performance (both AUC 0.74). Addition of other vulnerability measures yielded negligible incremental predictive information.

### Comparison to other literature

Other studies on 28-day mortality in primary care patients with COVID-19 aged 70 years or older are scarce, but our observed 28-day mortality fraction of 8.7% seems comparable to a study among community dwelling Italian COVID-19 patients aged 75 years or older, of whom 13% died [31]. As expected, the observed mortality in primary care is lower compared to nursing home, hospitalized or intensive care unit (ICU)-admitted COVID-19 patients, in whom mortality fractions between 19 and 37% have been reported [31, 32]. Of note, the 8.7% mortality in our study is among individuals with a diagnosis registration in primary care records, so including individuals who had severe enough symptoms to contact a medical doctor or have a COVID-19 test performed at the public health services. Mortality among the total general population of older individuals with COVID-19 may thus have been lower.

Comparing the predictive value of age and sex in older individuals with COVID-19 to the predictive value in a younger population shows a striking similarity. A previous study using the same data sources included all adult COVID-19 patients, predicting hospitalization instead of mortality [4]. This previous study found an AUC of 0.68 with unpenalized models using only age and sex for prediction of 30-day hospitalization (occurring in 6.8% of the study population), which is similar to the AUC of the basic model in our study [4]. It demonstrates that besides sex, *chronological* age remains quite discriminative for poor prognosis, even in a study sample of patients aged 70 years or older. This is further supported by the observation that even the best performing vulnerability measures only moderately increased the AUC in our study. So, even though we had expected that vulnerability measures, as an indicator of *biological* age, would have been more predictive, our study results do not clearly support this. It could imply that biological age was not as predictive for COVID-19 mortality as assumed, or that the vulnerability measures based on routine primary care records did not sufficiently capture it.

We have not identified studies on age- or sex-adjusted predictive value of frailty or vulnerability for COVID-19 mortality in older primary care patients, but studies on *hospitalized* COVID-19 patients show conflicting results. Three meta-analyses and two additional studies on early pandemic hospitalized COVID-19 patients (including 2020 data) reported an association between frailty measures such as the CFS and poor prognosis, although estimates from some individual studies were unadjusted for age or sex [7, 33–36]. On the other hand, more in line with our findings, a recent meta-analysis found that frailty (measured by different scales including the CFS) was *not* associated with short-term mortality, even when stratifying the included studies according to mean study population age [37]. This study also found that frail patients were less likely to be admitted to the ICU or to receive invasive mechanical ventilation, but have *higher* ICU-survival compared to non-frail patients, suggesting that the frail COVID-19 patients in hospital settings represent a select population after triage [37]. As decisions for hospital referral or intensive care admission are likely influenced by frailty status, comparisons with studies on hospitalized patients should be made with caution. Also, the hospital studies used different methodology and other frailty measures (e.g. the CFS), often based on clinician’s assessment and/or questionnaires, which differ from the vulnerability measures based on routine-care data in this study.

We found no incremental prognostic value for the FI in older COVID-19 patients in primary care, even though the same FI was found predictive of mortality in multiple general population-based studies in older people (thus without any selection on COVID-19 or acute illness), including also populations from primary care [15, 17, 38]. A possible explanation might be that during the peak stress-test of an acute illness, some cumulative deficits included in frailty indices like the FI used in this study – such as dependency on others, hearing impairment or social isolation – may be less important predictors of *short-term* prognosis of this severe acute disease compared to organ function-affecting comorbidities [16]. Indeed, the latter are more prominently embedded in the CCS and CCI, possibly explaining why the CCS and CCI had slightly better incremental predictive value compared to the FI.

We cannot derive from the current study which specific comorbidities would have most incremental predictive value, as we have only assessed this for the composite scores. Baseline comparisons (Table 1) suggest associations with mortality for all comorbidities except liver disease and cancer, yet univariable. Two meta-analyses and one individual study in hospital settings found univariable associations between short-term COVID-19 mortality and dementia, kidney disease, several cardiovascular diseases, diabetes, stroke or delirium, but no associations with chronic respiratory disease, cancer, smoking or obesity [33, 36, 37]. These associations should be interpreted with caution, because they are univariable and because comorbidities (e.g. respiratory disease) may have affected the likelihood of hospital referral. In the primary care setting, one study found that cardiovascular disease and diabetes were predictive of hospitalization (adjusted for age and sex) amongst COVID-19 patients, with the more cardiovascular diagnoses, the higher the risk [4]. Indeed, it may be possible that the number of cumulative comorbidities is more predictive than individual comorbidities. More insights on how comorbidities best predict COVID-19 mortality beyond age and sex still need to be obtained.

Lastly, it would be interesting to know whether our results are COVID-19 specific, or may also translate to other acute infections, such as pneumonia or influenza. We have not identified literature that validates predictive performance of vulnerability measures such as the FI in primary care patients with such acute respiratory infections. However, in line with our results, two large population-based studies on patients hospitalized for pneumonia found that routine care-based vulnerability measures (including a FI and the CCI) had very limited incremental predictive value for mortality besides age and sex, and only very high CFS scores were associated with increased mortality in pneumonia patients admitted to the ICU [39, 40]. Nevertheless, we believe more research is needed to understand whether it is the FI that we used in this study specifically, or if other measures reflecting a broader definition of frailty (such as the CFS) would also perform poorly in predicting short-term mortality after acute infections in primary care. The concept, though, that the short-term prognosis of older individuals with an acute illness is perhaps more driven by impaired organ functioning rather than social determinants of vulnerability may hold a promising avenue for future research and may in fact be one of the lessons we can learn from post-pandemic analyses like this study.

### Strengths and limitations

This study included a large sample of older COVID-19 patients in primary care that are representative of the general older population, instead of a hospital population already selected (possibly on frailty status) by referral from primary care. Follow-up was complete for nearly all patients, with high quality data on the outcome mortality. Further strengths of this study include robust modelling with non-linear terms for continuous predictors, the use of multiple metrics to evaluate incremental predictive value and the direct comparison between multiple vulnerability measures.

Our results should also be interpreted in the light of some limitations. First, selection bias could have occurred during the early pandemic when COVID-19 cases were often based on a clinical suspicion without confirmation by a test, or later if COVID-19 positive (self-)tests were not registered in primary care medical records. This could have led to overestimation of mortality, if only the most severely ill patients obtained a COVID-19 diagnosis registration, or to underestimation of mortality, if some of the included patients had (milder) respiratory infections other than COVID-19 (which we deem less likely given the lockdown situation). Second, due to the use of routine care data, predictor values may have been influenced by unregistered diagnoses, which may have led to underestimation of the FI and comorbidity scores and thereby underestimation of their predictive value if missing diagnoses affect patients with higher scores the most. We also used a slightly adjusted version of the CCI [16] as some items are not directly extractable from ICPCs (such as portal hypertension), which may have similarly underestimated predictive value of the CCI. Third, we have only evaluated six specific vulnerability measures available from routine-care data and therefore cannot generalize results to more broad-scope measures of frailty or its general concept. Fourth, we could not take into account COVID-19 viral strain type which may warrant caution in the generalization of results to current COVID-19 patients. However, our sensitivity analysis suggested similar results for the first two waves in 2020 patients only, suggesting that differences in viral strains did not influence our findings substantially. Fifth, we could not study measures of COVID-19 severity (e.g., oxygen saturation, symptoms) in this routine-care data based study, which may also provide prognostic information in real-world settings. Similarly, we could not take into account preventive or patient management (e.g., vaccinations, hospital referral or COVID-19 treatment) into our prognostic models, which might have also affected the outcome and hence the prognostic value of the different vulnerability measures [41].

### Implications

Our results imply that, even in older patients, age and sex (and to a lesser extent combined with somatic comorbidities reflecting impaired organ functioning) are predictive of short-term COVID-19 mortality risk in primary care. However, the routine care based FI may not inform on mortality risk beyond predictive information that is already captured by age and sex. So, when it comes to triage in the context of assigning limited health care resources to patients with the best prognosis, the role of routine care-based vulnerability measures (on top of age and sex) in identifying primary care patients with the highest mortality risk might not be as straightforward as we assumed at the beginning of the COVID-19 pandemic. The results of this study suggest that underlying comorbidities might be more important prognostic factors in the primary care setting.

Future research is needed to shed light on the generalizability of our results to other vulnerability measures and in the context of other acute infectious illnesses. It would, for example, be interesting to evaluate measures encompassing a broader definition of the (rather complex) frailty concept, such as the CFS, also in the primary care setting. To investigate whether predictive value of vulnerability measures could have been influenced by triage decisions, such studies could account for patient management decisions in the assessment of predictive value of vulnerability [41]. Moreover, we would encourage replication of the comparison in incremental predictive value between comorbidity-based scores, other vulnerability measures and broad-scope frailty indicators in older COVID-19 patients, and also in patients with other acute infectious illnesses (e.g., influenza). This would provide useful information for future pandemic preparedness and for better understanding the factors that are predictive of differential resilience of older patients with acute illnesses.

## Conclusions

When predicting 28-day mortality in older COVID-19 patients in the general population, this study found that age and sex remain good predictors. Although we found some increased predictive performance with addition of the CCI or CCS (both AUC 0.74, versus AUC 0.69 using age and sex only), the FI had no incremental predictive value (AUC 0.69). Therefore, in future pandemics or acute illnesses, caution is warranted in readily applying routine-care based vulnerability measures to predict mortality in primary care patients. Future studies are needed to evaluate whether other more broad-scope frailty indicators could still be useful in rapidly identifying (future pandemic) primary care patients with an acute infection at high risk of poor outcomes, or to confirm that we should indeed focus more on age, sex and comorbidities affecting vital organ function as short-term prognostic factors.

### Supplementary Information

**Supplementary Material 1.**

## Data Availability

Data used for the current study are not publicly available due to patient privacy. R scripts are available from the corresponding author upon reasonable request.

## Abbreviations

AHA AMC: Academic General Practitioner’s Network at Academic Medical Center Amsterdam
ANH VUMC: Academic Network of General Practice at VU University Medical Center in Amsterdam
ATC: Anatomical Therapeutic Chemical
AUC: Area under the receiver operating characteristic curve
CCI: Charlson Comorbidity Index
CCS: Chronic Comorbidity Score
CFS: Clinical Frailty Scale
CI: Confidence interval
COPD: Chronic obstructive pulmonary disease
eGFR: estimated Glomerular Filtration Rate
FI: Frailty Index
ICPC: International Classification of Primary Care
IPA: Index of prediction accuracy
IQR: Interquartile range
JGPN: Julius General Practitioner’s Network
TRIPOD: Transparent Reporting of a multivariable prediction model for Individual Prognosis Or Diagnosis
