# Supplementary material for: Limited incremental predictive value of the frailty index and other vulnerability measures from routine care data for mortality risk prediction in older patients with COVID-19 in primary care
Source: BMC Prim Care. 2024 Feb 23;25:70. doi: 10.1186/s12875-024-02308-5 (PMC10885372; doi:10.1186/s12875-024-02308-5)
Supplement: Supplementary file 1 — Supplementary Material 1. [file 12875_2024_2308_MOESM1_ESM.pdf]

Appendices

These Appendices belong to the article "**Limited incremental predictive value of the Frailty Index and other vulnerability measures from routine care data for mortality risk prediction in older patients with COVID-19 in primary care**" authored by Hannah la Roi-Teeuw and colleagues.

Content overview:

Appendix 1: Definitions of vulnerability measures page 2

Table S1. Frailty Index

Table S2. Charlson Comorbidity Index

Table S3. Chronic Comorbidity Score

Details on other predictors

Appendix 2: Details on methodology page 6

Details on multiple imputation

Details on sample size

Appendix 3: Supplementary results page 7

Table S4. Baseline characteristics per database

Table S5. Model coefficients

Table S6. Additional model fit and performance statistics

Table S7. Distribution of predicted risks

References page 12

Appendix 1: Definitions of vulnerability measures

The frailty index (FI) was used as defined in the original publication by Drubbel and colleagues.[1] In the current study, the FI was operationalized based on the ICPCs in Table S1.

Table S1. Frailty Index

Deficit name	ICPC (ever before)	ICPC within last 365 days	Points
General complaints	A28, B28, D28, F28, H28, K28, L28, N28, P28, R28, S28, T28, U28, X28, Y28, Z28	A01, A04, A05, B80, B81, B82, P78	0.028
Neoplasm - other	A79, B72, B73, B74, D74, D76, D77, F74, H75, K72, L71, N74, R84, S77, T71, U75, U76, U77, X75, X76, X77, Y78	-	0.028
Incontinence	D17, U04, X87	-	0.028
GI / Liver disease	D72, D97, D75, D94	D85, D86	0.028
Oesophagus disease	-	D84	0.028
Visual impairment	F83, F94, F84, F93	-	0.028
Cataract	F92	-	0.028
Hearing impairment	H84, H85, H86	-	0.028
Respiratory problems	-	K02, R02, R81	0.028
Angina pectoris	-	K74	0.028
Myocardial disease	K76	K75	0.028
Heart failure	K77	-	0.028
Atrial fibrillation/flutter	-	K78	0.028
Hypertension - uncomplicated	-	K86	0.028
Hypertension - complicated	K87	-	0.028
Dizziness	-	A06, H82, K88, N17	0.028
TIA / CVA	K90	K89	0.028
Vascular disease	K91, K92, K99	K93, K94	0.028
Fracture / Osteoporosis	L95	A80, L72, L73, L74, L75, L76	0.028
Arthritis / Osteoarthrosis	L88, L89, L91	-	0.028
Osteoarthrosis knee	L90	-	0.028
Neurologic disease	N86, N99, N87, N88, N94	N89	0.028
Depression	-	P03, P76	0.028

Sleep disturbance	-	P06	0.028
Cognitive impairment	P85, P70	P20	0.028
Psychiatric problems / Substance abuse	P72, P15, P17	P71, P73, P74, P16, P18, P19	0.028
COPD	R91, R95	-	0.028
Asthma	R96	-	0.028
Skin problems	S91	S70, S97	0.028
Weight problems	T83, T82	T05, T07, T08	0.028
Thyroid disorders	-	T85, T86	0.028
Diabetes mellitus	T90	-	0.028
Urinary disease	U99	-	0.028
Prostate problems	Y77, Y85	-	0.028
Social problems	Z15	Z01, Z03, Z04, Z29, Z12, Z14	0.028
Polypharmacy	-	(see definition number of chronically prescribed drugs below)	0.028

GI = gastro-intestinaal, TIA = transient ischemic accident, CVA = cerebral vascular accident, COPD = chronic obstructive pulmonary disease

The Charlson Comorbidity Index (CCI) was calculated using the ICPCs in Table S2 (method adapted from Swart and colleagues[2]).

Table S2. Charlson Comorbidity Index

**Category**	**ICPC (ever before)**	**Points***
Age $\geq$ 70	-	3
Age $\geq$ 80	-	1
Myocardial infarction	K75, K76.02	1
Congestive heart failure	K71.02, K77, K84.03, K77.01, K77.02	1
Peripheral vascular disease	K91, K92, K99.01, K92.01, K92.02, K92.03	1
Cerebrovascular disease	K89, K90, K90.01, K90.02, K90.03	1
Dementia	ICPC P70	1
Chronic pulmonary disease	K82, R91, R95, R91.01, R91.02	1
Rheumatic disease	L88, L99.12	1
Peptic ulcer disease	D86	1
Mild liver disease	D72, D97, D72.01, D72.02, D72.03, D72.04, D72.05, D97.04	1
Moderate or severe liver disease	D97.05	2
Diabetes without chronic complication	T90, W84.02, T90.01, T90.02	1

Diabetes with chronic complication	F83.01, N94.02, K99.06, S97.03	1
Hemiplegia or paraplegia	N18	2
Renal disease	U88, U99.01	2
Any malignancy, including lymphoma and leukemia, except malignant neoplasm of skin	B72, B73, B74, D74, D75, D76, D77, F74.01, H75.01, K72.01, L71, N74, R84, R85, T71, U75, U76, U77, W72, X75, X76, X77, Y77, Y78, B72.01, B72.02, B74.01, X76.01, X77.01, X77.02, Y78.01, Y78.02, Y78.03	2
Metastatic solid tumor	A79	4
AIDS/HIV	B90, B90.01, B90.02	6

*Points are cumulative. For example, a 83 years-old patient scores "Age>70" and "Age>80" (3+1 points), a patient with metastatic solid tumor scores "Any malignancy" and "Metastatic solid tumor" (2+4 points).
AIDS = acquired immune deficiency syndrome, HIV = human immunodeficiency virus

The Chronic Comorbidity Score (CCS) was calculated according to the definitions by Herings and colleagues, see Table S3 below.[3]

Table S3. Chronic Comorbidity Score

Domain	ICPC/ATC/code (ever before)	ICPC/ATC/code within last [period]	Points
Cardiovascular	K74, K74.01, K74.02, K75, K76, K76.01, K76.02, K77, K77.01, K77.02, K78, K89, K90, K90.01, K90.02, K90.03, K91, K86, K87, K79, K79.01, K79.02, K73, K73.01, K73.02, K84, K84.01, K84.02, K84.03, K84.07, K92, K92.01, K92.02, K92.03, K80, K80.01, K80.02, K80.03, K82		1
Diabetes	T90, T90.01, T90.02		1
Neurological	P70, N87, N87.01, N88, N86, N99, N99.01, N99.02, N99.03		1
Heart valve	K71, K71.02, K83, K83.01, K83.02		1
Liver	D97, D97.04, D72, D72.04, D72.05	D97.05 [5 years], D72.01-3 [6 months]	1
Lung	R95, R96, R96.02, R91, R91.01, R91.02, K93, R70, R82, R89, R99, R99.06, R99.10	2209 [2 years], 3549 [1 year], H02AB06 [2 years], H02AB07 [2 years]	1

Cancer	X76, X76.01, Y78, Y78.01-3, N74, U76, T71, D76, D74, W72, U77, U75, D75, A79, X75, R84, B74, B74.01, X77, X77.01-2, S77, S77.01-4, R85, R86, B72, B72.01-2, B73		1
Kidney	U85, U85.01, U88, U99, U99.01-3	3583 [1 year], 1919 [1 year], 1918 [1 year], 524 [1 year]	1
Immune system	B90, B90.01-2, T99.01	H02A* [6 months], L01* [6 months], L04A* [6 months]	1

Details on other predictors

The number of chronically prescribed medications was defined as the number of unique registered ATC codes (excluding COVID-19 vaccinations) prescribed within the last year which are either (1) indicated as ‘chronic’ medication in the primary care records or (2) prescribed at least three times within the last year, of which at least once within the last six months before index.[1]

Cognitive impairment was defined as the presence of at least one diagnosis of a cognitive disorder, based on ICPCs P20 (Memory / concentration / orientation disturbance), P85 (Mental retardation) and P70 (Dementia / Alzheimer’s disease).

Renal function was defined as the last available eGFR, which could be either quantitative or qualitative (e.g., “>90”).

Appendix 2: Details on methodology

Details on imputation methods
Multiple imputation with chained equations was performed using the *mice* package in R. Missing values on categorical eGFR (5.5%) were imputed using polytomous regression. The following binary variables were used as predictors for imputation: sex, 28-day mortality, 90-day mortality, (history of) cardiovascular disease, hypertension, heart failure, coronary artery disease, cerebrovascular disease, peripheral artery disease, atrial fibrillation, heart valve disease, lung disease, chronic pulmonary obstructive disease, asthma, dementia, cognitive impairment, neurological disease, solid cancer, hematological cancer, skin cancer, diabetes, liver disease, chronic kidney disease, any kidney disease, immunocompromised status. The following categorical variables were used as predictors: social economic status (three categories, based on relative income) and database. The following continuous variables were used as predictors: age (restricted cubic splines with four knots), the interaction between sex and all spline terms of age, FI, number of chronically prescribed drugs, CCI and CCS. A total of ten datasets were imputed. Rubin's rules were used for pooling of performance metrics. For pooling of coefficients, the average coefficient was calculated over all imputed datasets which selection of the variable during penalized regression if the variable was selected in at least five datasets (majority vote), otherwise the coefficient was considered dropped.

Details on sample size
Sample size calculations for different scenario's were performed a priori to scope the feasibility of the study (see protocol).[4] Final calculations were in line with these approximations: the available total sample size, the c-statistic of the basic model and the observed 28-day mortality fraction, allowed for up to 14 candidate predictor parameters to ensure robust models with a maximum shrinkage of 10% (*pmsampsize* R package) [5]. Hence all anticipated models could be fitted. The sensitivity analysis on a restricted cohort of only individuals with a COVID-19 diagnosis in 2020 (n = 2,362), before the start of the vaccination campaigns, was deemed too small to have a valid interpretation of performance metrics (at risk of overfitting or underfitting); hence results of this exploratory analysis were only scoped for trends and not formally published here.

Appendix 3: Supplementary results

Table S4. Baseline characteristics per database

		Database		
**Total study population (n=4,065)**		**AHA AMC (n=1,107)**	**JGPN (n=1,417)**	**ANH VUMC (n=1,541)**
Demographics				
Age, median [IQR]	77 [73, 83]	76 [73, 82]	78 [73, 83]	77 [73, 82]
Female, n (%)	2185 (53.8)	602 (54.4)	770 (54.3)	813 (52.8)
Comorbidities, n (%)				
Cardiovascular disease	2898 (71.3)	794 (71.7)	1046 (73.8)	1058 (68.7)
Hypertension	2121 (52.2)	575 (51.9)	792 (55.9)	754 (48.9)
Heart failure	475 (11.7)	123 (11.1)	200 (14.1)	152 (9.9)
Coronary artery disease	964 (23.7)	246 (22.2)	377 (26.6)	341 (22.1)
Cerebrovascular disease	662 (16.3)	189 (17.1)	248 (17.5)	225 (14.6)
Peripheral artery disease	349 (8.6)	96 (8.7)	136 (9.6)	117 (7.6)
Atrial fibrillation	621 (15.3)	174 (15.7)	235 (16.6)	212 (13.8)
Diabetes	1383 (34.0)	388 (35.0)	498 (35.1)	497 (32.3)
Pulmonary disease	1072 (26.4)	294 (26.6)	399 (28.2)	379 (24.6)
COPD	493 (12.1)	107 (9.7)	202 (14.3)	184 (11.9)
Asthma	507 (12.5)	143 (12.9)	191 (13.5)	173 (11.2)
Chronic kidney disease	1061 (26.1)	276 (24.9)	332 (23.4)	453 (29.4)
Liver disease	138 (3.4)	58 (5.2)	31 (2.2)	49 (3.2)
Dementia	193 (4.7)	46 (4.2)	87 (6.1)	60 (3.9)
Immuno-compromised*	526 (12.9)	150 (13.6)	183 (12.9)	193 (12.5)
Cancer	1129 (27.8)	303 (27.4)	422 (29.8)	404 (26.2)
Vulnerability measures				
Frailty index, median [IQR]	0.30 [0.22, 0.38]	0.28 [0.20, 0.36]	0.30 [0.22, 0.38]	0.30 [0.20, 0.38]
Chronic Comorbidity Score, median [IQR]	2 [1, 3]	2 [1, 3]	2 [1, 3]	2 [1, 3]
Charlson Comorbidity Index, median [IQR]	5 [4, 7]	5 [4, 7]	5 [4, 7]	5 [4, 7]
Number of drugs, median [IQR]	4 [1, 7]	4 [2, 8]	4 [2, 7]	3 [1, 6]
eGFR				
<15	18 (0.5)	4 (0.4)	5 (0.4)	9 (0.6)
15-30	124 (3.2)	30 (2.9)	55 (4.1)	39 (2.7)
30-45	353 (9.2)	98 (9.4)	132 (9.8)	123 (8.5)
45-60	746 (19.4)	217 (20.8)	254 (18.8)	275 (19.0)

60-75	1221 (31.8)	356 (34.1)	439 (32.5)	426 (29.4)
75-90	1112 (28.9)	276 (26.5)	390 (28.8)	446 (30.8)
>90	268 (7.0)	62 (5.9)	77 (5.7)	129 (8.9)
Unknown	223 (5.5)	64 (5.8)	65 (4.6)	94 (6.1)
Cognitive impairment, n (%)	627 (15.4)	185 (16.7)	242 (17.1)	200 (13.0)

*According to a diagnosis of immunodeficiency or use of immunosuppressants, including prednisone, biologicals and oncolytics.

AHA AMC = Academic General Practitioner's Network at Academic Medical Center Amsterdam, JGPN = Julius General Practitioner's Network, ANH VUMC = Academic Network of General Practice at VU University Medical Center in Amsterdam, IQR = interquartile range, COPD = chronic obstructive pulmonary disease, eGFR = estimated glomerular filtration rate

Table S5. Model coefficients

Variable*	Basic	Basic + FI	Basic + drugs	Basic + CI	Basic + CCI	Basic + CCS	Basic + eGFR
Intercept	-8.478	-8.769	-8.252	-8.292	-7.287	-8.025	-7.922
Age	0.080	0.078	0.075	0.077	0.037	0.062	0.075
Age'	0.020	0.020	0.023	0.017	0.029	0.028	0.013
Age''	.	.	.	.	0.025	0.003	.
Sex	-0.441	-0.483	-0.453	-0.462	-0.472	-0.538	-0.484
Sex:age	-0.003	-0.003	-0.003	-0.003	-0.003	-0.002	-0.003
Sex:age'	.	.	.	.	.	.	.
Sex:age''	.	.	.	.	.	.	.
FI		1.730					
FI'		.					
FI''		-5.540					
Drugs			0.017				
Drugs'			0.013				
Drugs''			0.093				
CI				0.505			
CCI					0.376		
CCI'					-0.004		
CCI''					-0.280		
CCS						0.394	
CCS'						.	
CCS''						-0.427	
eGFR 15-30							0.645
eGFR 30-45							0.164
eGFR 45-60							.
eGFR 60-75							-0.327
eGFR 75-90							-0.414
eGFR >90							-0.153

FI = frailty index, drugs = number of chronically prescribed drugs, CI = cognitive impairment, CCI = Charlson Comorbidity Index, CCS = Chronic Comorbidity Score, eGFR = estimated glomerular filtration rate

*Variables with accents indicate spline terms. For sex the reference category was male, for CI the reference category was no cognitive impairment, for eGFR the reference category was eGFR <15. A dot indicates that the variable was used as candidate predictor in the model, but dropped by variable selection in the elastic net penalization.

Table S6. Additional model fit and performance statistics

	Basic (age + sex + age*sex)	Basic + Frailty index	Basic + Cognitive impairment	Basic + eGFR	Basic + Number of drugs	Basic + CCI	Basic + CCS
Unpenalized logistic regression							
AIC	2236	2234	2223	2217	2217	2164	2173
LRT* χ^2	n/a	8.4	14.7	31.4	24.9	77.9	68.9
p-value	n/a	0.038	<0.001	<0.001	<0.001	<0.001	<0.001
Apparent performance (penalized logistic regression)							
AUC (95% CI)	0.68 (0.65, 0.71)	0.68 (0.65, 0.71)	0.70 (0.67, 0.73)	0.70 (0.67, 0.73)	0.70 (0.67, 0.73)	0.74 (0.71, 0.76)	0.74 (0.71, 0.76)
IPA (95% CI)	2.8 (-2.8, 14.4)	2.8 (-2.2, 15)	3.5 (-2.6, 14.1)	3.5 (-7.0, 14)	3.5 (-2, 14.1)	4.7 (-0.5, 14.8)	4.9 (-1.3, 14.8)
Performance (internal validation of penalized logistic regression)							
Calibration slope (95% CI)	1.8 (1.5, 2.1)	1.6 (1.3, 1.9)	1.9 (1.6, 2.1)	1.7 (1.4, 2.0)	1.7 (1.5, 2.0)	1.7 (1.5, 2.0)	1.7 (1.5, 2.0)

* Model is tested compared to the basic model.

CCI = Charlson Comorbidity Index, CCS = Chronic Comorbidity Score, eGFR = estimated glomerular filtration rate, AIC = Akaike's information criterion, LRT = likelihood ratio test, AUC = area under the curve, CI = confidence interval, IPA = index of prediction accuracy

Table S7. Distribution of predicted risks

Predicted risk (%)	Minimum	Q1	Median	Q3	Maximum
Basic (age + sex + age*sex)	6.6	7.0	7.6	9.4	25.9
Basic + Frailty index	6.6	7.0	7.6	9.4	25.9
Basic + Number of drugs	5.9	6.7	7.5	9.6	30.4
Basic + Cognitive impairment	5.9	6.8	7.5	9.6	31.5
Basic + CCI	5.0	6.0	7.5	10.2	41.1
Basic + CCS	4.3	6.1	7.6	10.1	32.3
Basic + eGFR	5.9	6.6	7.5	9.7	31.0

CCI = Charlson Comorbidity Index, CCS = Chronic Comorbidity Score, eGFR = estimated glomerular filtration rate,

References

1 Drubbel I, De Wit NJ, Bleijenberg N, *et al.* Prediction of adverse health outcomes in older people using a frailty index based on routine primary care data. *J Gerontol A Biol Sci Med Sci*. 2013;68:301–8.

2 Swart K, Van Der Heijden AA, Blom M, *et al.* Identification of frailty in primary care: accuracy of electronically derived measures. *British Journal of General Practice*. 2023;73:e752–9.

3 Herings RMC, Swart KMA, Van Der Zeijst BAM, *et al.* Development and validation of an algorithm to estimate the risk of severe complications of COVID-19: a retrospective cohort study in primary care in the Netherlands. *BMJ Open*. 2021;11:e050059.

4 la Roi-Teeuw H, Luijken K, Blom M, *et al.* Incremental predictive value of different frailty proxy measures for mortality risk prediction in older patients with COVID-19 infection in primary care. OSF. 2023. https://doi.org/10.17605/OSF.IO/E2H8K

5 Ensor J, Martin EC, Riley RD. Package 'pmsampsize' Title Calculates the Minimum Sample Size Required for Developing a Multivariable Prediction Model. Published Online First: 2022. doi: 10.1002/sim.7992
